# Cost-effectiveness of statins, berberine, and combination for primary cardiovascular disease prevention in Scotland

**DOI:** 10.1038/s44325-026-00121-w

**Published:** 2026-04-23

**Authors:** Yuanqing Xia, Kathy Leung, Jie V. Zhao

**Affiliations:** 1 School of Public Health, Li Ka Shing Faculty of Medicine, The University of Hong Kong, Patrick Manson Building, Hong Kong SAR, People’s Republic of China; 2 The Hong Kong Jockey Club Global Health Institute, Hong Kong SAR, People’s Republic of China; 3 WHO Collaborating Centre for Infectious Disease Epidemiology and Control, School of Public Health, LKS Faculty of Medicine, The University of Hong Kong, Hong Kong SAR, People’s Republic of China; 4 Laboratory of Data Discovery for Health Limited (D24H), Hong Kong Science Park, Hong Kong SAR, People’s Republic of China; 5 The University of Hong Kong-Shenzhen Hospital, Shenzhen, People’s Republic of China; 6 State Key Laboratory of Pharmaceutical Biotechnology, The University of Hong Kong, Hong Kong SAR, People’s Republic of China

**Keywords:** Cardiology, Diseases, Health care, Medical research

## Abstract

We aim to estimate and compare the cost-effectiveness of statins, berberine, and their combined use for primary cardiovascular disease (CVD) prevention. The Scottish CVD Policy Model was used to predict long-term health and cost outcomes in Scottish adults aged 40 years or older without pre-existing CVD. Intervention and cost inputs were sourced from published literature and health service cost data. The primary outcome measure was the lifetime incremental cost-effectiveness ratio (ICER), evaluated as cost per quality-adjusted life year (QALY) gained. Five strategies were analyzed for individuals with ASSIGN risk scores ≥20% and ≥10%: no intervention, atorvastatin 20 mg/day, berberine 1000 mg/day, simvastatin 20 mg plus berberine 1500 mg/day, and simvastatin 20 mg plus berberine 900 mg/day. All intervention strategies were cost-effective, compared to no intervention, at the threshold of ICER of £20,000 per QALY. Compared to statins, berberine was less cost-effective, but the combined interventions remained cost-effective. Notably, when using drug costs from China (reflecting lower berberine prices), berberine and the combined interventions were preferable to statins alone. Statins, berberine, and combined interventions are all cost-effective options for primary CVD prevention. Berberine could be considered a valuable alternative or complementary therapy, particularly if its price decreases below that of statins.

## Introduction

Cardiovascular disease (CVD) is the leading cause of morbidity and mortality worldwide, which poses a heavy burden on healthcare^1^. The primary prevention of CVD through the management of dyslipidemia, a major risk factor for CVD, is a critical strategy for improving population health outcomes and reducing the associated economic impact. Statins (HMG-CoA reductase inhibitors) are lipid-lowering drugs used as a cornerstone treatment for the primary and secondary prevention of CVD^2–4^. Statin use has increased substantially in recent years^5,6^, with nearly two-thirds of those taking statins for primary prevention^7^. The clinical guidelines also recommend statin use among people previously considered at low risk of CVD^4,8–10^. According to the seven European Society of Cardiology/European Atherosclerosis Society (ESC/EAS) guidelines, the proportion of patients eligible for statins increased from approximately 8% in 1987 to 61% in 2016 due to the change in guidelines^11^. However, statin intolerance occurs in as many as 9.1% of patients treated with statins^12^, due to side effects, such as myopathy, hepatotoxicity and incident diabetes^13–16^. Although several newer lipid‑lowering therapies, such as PCSK9 inhibitors, bempedoic acid, and inclisiran, provide substantial low-density lipoprotein cholesterol (LDL‑C) reductions, their high cost and restricted eligibility criteria may limit their accessibility for many patients.

In recent years, there has been growing interest in exploring natural, plant-derived compounds as alternative or complementary options for lipid-lowering^17,18^. Berberine, a widely accessible nutrient supplement in the United Kingdom (UK)^19^, is one of the promising candidates. Several recent meta-analyses of randomized clinical trials (RCTs) have provided compelling evidence for the lipid and lipoprotein-modulating effects of berberine alone and combined with statins^20–22^. Compared with the statins alone, berberine plus statins was more effective in lowering triglyceride and total cholesterol^22^. Previous studies also show that berberine is well-tolerated. Notably, unlike statins, which increase the risk of diabetes, berberine improves glucose metabolism^23,24^, and is used as a nutrient supplement to lower glucose. Given its favorable safety profile and lipid-lowering benefits, berberine has been proposed as a potential nutritional option for dyslipidemia, particularly among patients with statin intolerance. This perspective is supported by both the International Lipid Expert Panel^25^ and the 2019 ESC/EAS Guidelines^26^, categorizing berberine under “dietary supplements and functional foods”, and emphasizing the current lack of robust randomized trial evidence. A recent meta-analysis conducted in 2023 evaluated 44 RCTs comparing berberine alone or combined with statins versus statins or routine care for CVD^27^. The pooled data indicate that berberine significantly improves lipid profiles, neurological function, inflammation, and atherosclerosis in CVD treatment, with no serious adverse reactions noted. This suggests that berberine may be a promising alternative for CVDs.

However, the cost-effectiveness of berberine and berberine combined with statins is still unclear. We did not identify any studies comparing berberine, berberine combined with statins, versus statins in terms of overall cost-effectiveness. Considering the research gap, our study objective was to estimate and compare the cost-effectiveness of statins, berberine, and the combined intervention of statins and berberine for primary prevention of CVD based on 10-year CVD risk.

## Results

### The CVD risk profile of the final intervention-eligible population

Table S1 shows the CVD risk profile of the eligible population we included in the analysis. Women account for 55.6% of this population. On average, women had lower mean systolic blood pressure (SBP), higher mean total cholesterol (TC) level, and higher mean high-density lipoprotein cholesterol (HDL-c) level compared to men.

### Cost-effectiveness of different intervention strategies

**Fig. 1:**
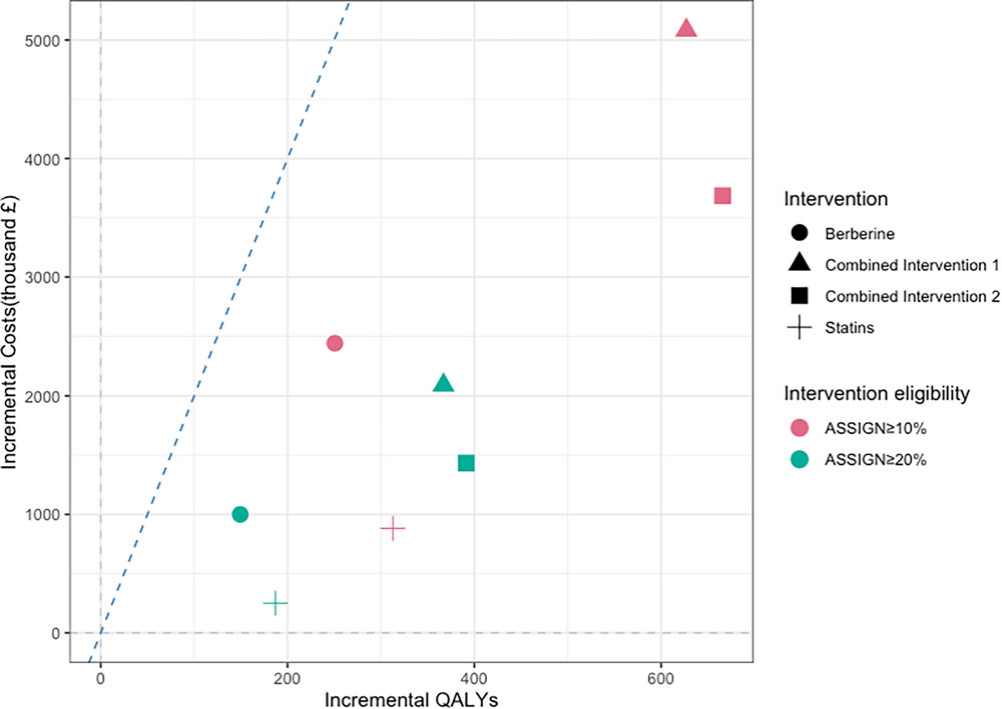
Cost-effectiveness plane for all intervention strategies. The plot shows the incremental cost-effectiveness ratios (ICERs) of all intervention strategies. The blue dashed line represents the cost-effectiveness threshold of £20,000/quality-adjusted life-year (QALY). An intervention strategy is considered cost-effective if the colorful point is below the blue dashed line.

Table 1 and Fig. 1 show the costs and quality-adjusted life-year (QALY) estimates for different intervention strategies in primary prevention, stratified by ASSIGN risk thresholds. Lowering the ASSIGN risk threshold from 20% to 10% increases the proportion of people in Scotland over 40 without CVD who are eligible for primary prevention intervention from 34.4% to 60.0%.

**Table 1:** The cost-effectiveness of statins, berberine, and combined interventions

Interventions	Sample size	Number treated	Primary CVD events prevented	NNT	Undiscounted life-years	Discounted QALYs gained	Discounted cost, £	ICER, £/QALY
**Intervention strategies comparable to no intervention**								
*Intervention eligibility: ASSIGN* ≥ *20%*								
No intervention	3869	0	Reference	Reference	Reference	Reference	Reference	Reference
Statins	3869	1331	50.2	26.5	253.2	187.1	251,271.6	1343.3
			(30.5 – 68.0)		(94.4 – 428.3)	(60.6 – 328.9)	(−94,248.8 – 582,182.7)	(−1221.2 – 1977.0)
Berberine	3869	1331	42.1	31.6	206.6	149.3	1,000,074.1	6697.3
			(28.4 ~ 53.4)		(76.4 ~ 336.1)	(47.5 ~ 251.4)	(658,657.2 – 1,303,043.9)	(4905.0 – 14,140.7)
Combined intervention 1	3869	1331	95.4	14	468.8	366.9	2,090,728.6	5697.8
			(63.9 – 120.9)		(160.2 – 764.3)	(124.9 – 598.1)	(1,237,065.1 – 2,841,177.9)	(4526.5 – 10,194.6)
Combined intervention 2	3869	1331	100.6	13.2	499.8	391.3	1,433,414.2	3662.9
			(64.1 – 132.0)		(183.4 – 822.3)	(144.8 –643.2)	(649,229.1 –2,174,503.1)	(2902.7 – 4930.7)
*Intervention eligibility: ASSIGN* ≥ *10%*								
No intervention	3869	0	Reference	Reference	Reference	Reference	Reference	Reference
Statins	3869	2323	89.5	26	431.1	312.7	882,016.8	2820.7
			(55.1 – 121.7)		(163.5 – 722.1)	(99.0 – 546.9)	(196,381.7 ~ 1,551,102.4)	(1595.8 ~ 3341.8)
Berberine	3869	2323	75.7	30.7	355.3	250.6	2,440,797.4	9739.1
			(51.8 – 95.4)		(142.5 – 568.1)	(82.2 – 418.8)	(1,811,308.8 – 3,005,828.0)	(6986.8 ~ 21,923.6)
Combined intervention 1	3869	2323	171.8	13.5	803.9	626.9	5,082,356.9	8107.4
			(117.6 – 215.7)		(294.1 – 1296.1)	(222.5 – 1014.3)	(3,483,097.2 – 6,490,100.3)	(6296.9 – 16,118.5)
Combined intervention 2	3869	2323	180.2	12.9	852.8	665.7	3,686,285.5	5537.7
			(118.0 – 234.3)		(331.9 – 1382.2)	(253.9 – 1083.8)	(2,184,066.0 ~ 5,129,760.2)	(4555.5 – 8498.6)
**Intervention strategies comparable to statin intervention**								
*Intervention eligibility: ASSIGN* ≥ *20%*								
Statins	3869	1331	Reference	26.5	Reference	Reference	Reference	Reference
Berberine	3869	1331	−8.1	31.6	−46.6	−37.7	748,802.6	Dominated^a^
			(−15.6 to -0.9)		(−127.5 – 23.2)	(−102.7 – 18.4)	(616,307.8 – 851,647.2)	
Combined intervention 1	3869	1331	45.2	14	215.6	179.9	1,839,457.1	10,225.9
			(31.7 – 55.0)		(13.0 – 393.4)	(19.8 – 320.2)	(1,277,716.0 – 2,325,393.8)	(6765.6 – 44,630.8)
Combined intervention 2	3869	1331	50.4	13.2	246.6	204.3	1,182,142.7	5786.8
			(32.9 – 64.3)		(83.1 – 401.0)	(75.3 – 325.3)	(715,032.4 – 1,609,393.2)	(4757.1 – 9830.4)
*Intervention eligibility: ASSIGN* ≥ *10%*								
Statins	3869	2323	Reference	26	Reference	Reference	Reference	Reference
Berberine	3869	2323	−13.8	30.7	−75.8	−62.1	1,558,780.6	Dominated^a^
			(−27.1 to -1.3)		(−210.5 – 40.9)	(−169.7 – 31.5)	(1,270,372.1 – 1,784,238.6)	
Combined intervention 1	3869	2323	82.3	13.5	372.8	314.2	4,200,340.1	13,368.8
			(58.7 – 100.3)		(27.9 – 668.2)	(38.7 – 553.9)	(3,149,543.4 – 5,067,201.1)	(8733.8 – 50,122.2)
Combined intervention 2	3869	2323	90.7	12.9	421.7	353	2,804,268.7	7944.6
			(60.8 – 115.8)		(145.5 – 677.6)	(139.4 – 555.1)	(1,926,017.0 –3,594,800.5)	(6381.7 – 14,105.8)

*CVD* Cardiovascular Disease, *NNT* the number needed to treat, *QALY* quality-adjusted life-year, *ICER* incremental cost-effectiveness ratio; ICER = Incremental cost/Incremental QALY.

^a^More expensive and less effective than the reference.

Compared to no intervention, at the threshold of £ 20,000 per QALY, all intervention strategies were found to be cost-effective in preventing primary CVD events. For individuals with ASSIGN ≥ 20%, statin intervention was estimated to prevent 50.2 CVD events and produce an incremental cost-effectiveness ratio (ICER) of £1343.3/QALY, berberine intervention was estimated to prevent 42.1 CVD events with an ICER of £6697.3/QALY, combined intervention 1 was estimated to prevent 95.4 CVD events with an ICER of £5697.8/QALY, and combined intervention 2 was estimated to prevent 100.6 CVD events with an ICER of £3662.9/QALY. For individuals with ASSIGN ≥ 10%, the corresponding estimates were 89.5 events (ICER £2820.7/QALY) for statins, 75.7 events (ICER £9739.1/QALY) for berberine, 171.8 events (ICER £8107.4/QALY) for combined intervention 1, and 180.2 events (ICER £5537.7/QALY) for combined intervention 2.

Compared to statin intervention, the berberine strategy was predicted to be more costly and less effective (i.e., dominated), but combined interventions remained cost-effective (combined intervention 1: ICER £10,225.9/QALY for ASSIGN ≥ 20%, ICER £13,368.8/QALY for ASSIGN ≥ 10%; combined intervention 2: ICER £5786.8/QALY for ASSIGN ≥ 20%, ICER £7944.6/QALY for ASSIGN ≥ 10%) at the threshold of £ 20,000 per QALY.

When using drug costs from China, where the price of berberine is reduced to below that of statins, all intervention strategies were still cost-effective compared to no intervention for both ASSIGN ≥ 20% and ASSIGN ≥ 10% thresholds. Berberine alone or in conjunction with statins was also found to be cost-effective compared to statins alone for both ASSIGN risk levels. (Table S2–S3 and Fig. S1)

### Probability of cost-effectiveness

**Fig. 2:**
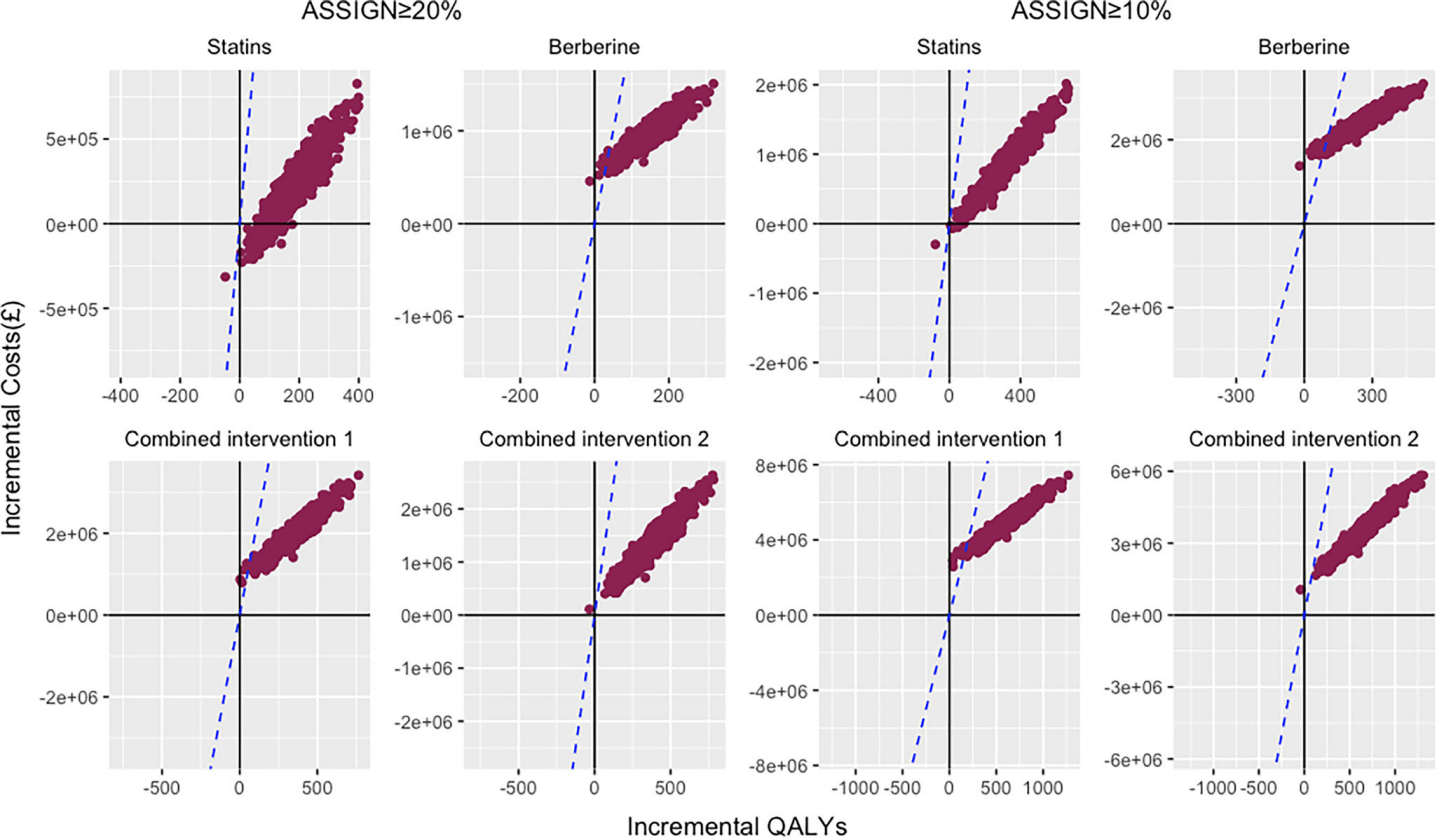
Probabilistic sensitivity analysis for all intervention strategies. This scatter plot shows the incremental cost-effectiveness ratios (ICERs) generated by 1000 Monte Carlo simulations for statins, berberine, and combined interventions. The blue dashed line represents the cost-effectiveness threshold of £20,000/quality-adjusted life-year (QALY). The proportions of dots below these blue dashed lines identify the likelihood that the Monte Carlo simulation results would yield an ICER below £20,000/QALY.

Probabilistic sensitivity analysis (PSA) (Fig. 2) showed that at cost-effectiveness thresholds below £20,000/QALY, all intervention strategies were cost-effective in most model iterations.

**Fig. 3:**
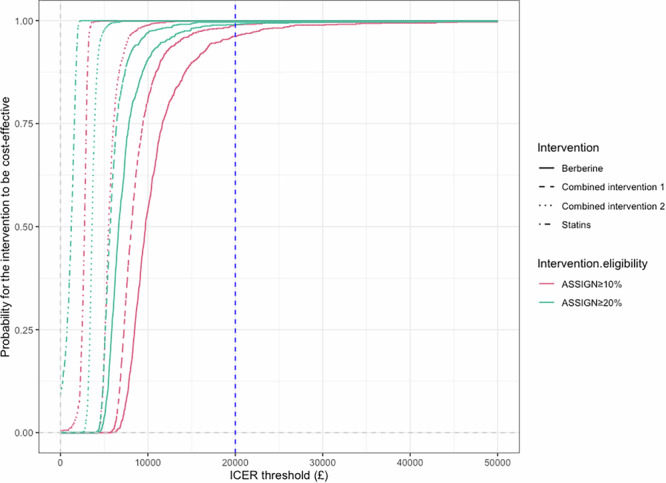
Cost-effectiveness acceptability curves for all intervention strategies. The plot illustrates the cost-effectiveness acceptability curves for all intervention strategies, showing the probability of being cost-effective at various willingness-to-pay thresholds. The blue dashed line represents the cost-effectiveness threshold of £20,000/quality-adjusted life-year (QALY). The probabilities of statins, berberine, and combined interventions being cost-effective are over 95% at a £20,000/QALY threshold.

Cost-effectiveness acceptability curves (Fig. 3) indicated that at a £20,000/QALY threshold, statins, berberine, combined intervention 1, and combined intervention 2 were the optimal strategies 99.9%, 99.0%, 99.6%, and 99.9% of the time, respectively, for individuals with ASSIGN risk ≥20%. For those with ASSIGN risk ≥10%, the corresponding optimal strategy probabilities were 99.9%, 96.2%, 98.9%, and 99.9%.

The scenario analysis, which assumed a reduced berberine price by switching to Chinese sources, yielded similar results (Figs. S2-S3).

### Deterministic sensitivity analysis

**Fig. 4:**
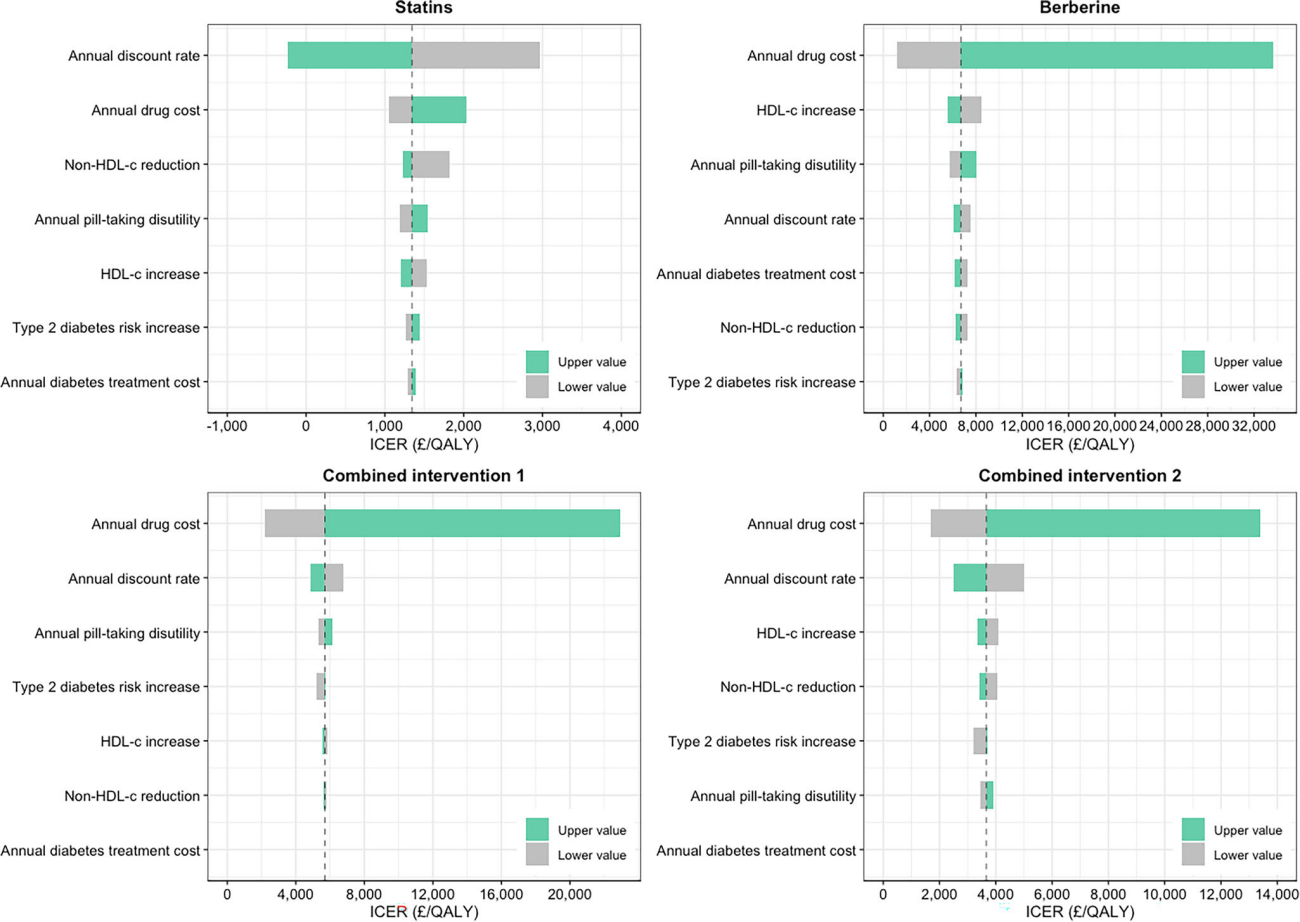
Tornado plots for the influential model measures for all intervention strategies among individuals with ASSIGN risk ≥20%. These tornado plots illustrate the impact of influential model parameters on the cost-effectiveness of statins, berberine, and combined interventions for individuals with an ASSIGN risk ≥ 20%. The dashed line represents the baseline cost-effectiveness value of statins, berberine, and combined interventions.

Tornado plots (Fig. 4 and Fig. S4) indicate that annual drug costs of statins and berberine and the annual discount rate had the largest impact on cost-effectiveness outcomes. For statin therapy, the discount rate was the most influential parameter, followed by drug cost. For berberine monotherapy, drug cost ranked highest, with HDL cholesterol increase and annual pill-taking disutility as secondary factors. As to combined interventions, drug cost again emerged as the most influential parameter, followed by discount rate.

## Discussion

This study provides a comprehensive cost-effectiveness analysis of various intervention strategies for the primary prevention of CVD. The analysis compared the cost-effectiveness of statin therapy, berberine supplementation, and combination approaches. The results indicate that compared to no intervention, statins, berberine, and combined interventions are all be cost-effective in preventing primary CVD events. When compared to statin intervention, berberine alone is less effective and more costly, but combined interventions remain cost-effective. However, if the price of berberine decreased to below that of statins, berberine alone or combined with statins was more cost-effective compared to statins monotherapy.

To our knowledge, this is the first comprehensive cost-effectiveness analysis examining berberine, both alone and in combination with statins, for primary CVD prevention. Previous studies have primarily focused on the cost-effectiveness of statins monotherapy or simplistic comparisons of lipid-lowering medications versus supplements^28–33^. This analysis provides a more robust assessment of the healthcare costs and QALYs associated with different intervention strategies. Compared to no intervention, statins, berberine, and combined interventions all demonstrate cost-effectiveness, indicating that high-risk populations have multiple options for the primary prevention of CVD events. For patients with hyperlipidemia who are intolerant to statins or seeking to optimize their lipid profiles through natural means, berberine offers an alternative as a nutraceutical, which can be used alone or in combination with statins. However, berberine is less cost-effective compared to statin intervention, while combined interventions remain cost-effective. This suggests that for patients with hyperlipidemia who tolerate statins, either statins alone or in combination with berberine is still the superior choice for primary prevention.

Our results confirm the cost-effectiveness of berberine, but it is less cost-effective than statins alone in the setting of Scotland. Conversely, it would be more cost-effective in the setting where we used the cost in China. The UK’s healthcare system, primarily driven by the National Health Service (NHS), offers free medical services to residents and applies strict price controls on prescription medications, resulting in low statin prices^34^. However, berberine, categorized as a dietary supplement, is not covered by the NHS, and its price is determined by market supply and demand. In Scotland, berberine needs to be imported from overseas, which increases costs related to transportation and tariffs. Additionally, the relatively low demand and limited competition contribute to its higher price. In contrast, in China, berberine is widely used in traditional Chinese medicine, leading to high market demand and intense competition^35,36^. There are numerous berberine cultivation bases and production factories, resulting in lower production and transportation costs, and consequently, lower prices. If Scotland were to include berberine in the NHS coverage or reduce its price to below that of statins, the strategies of using berberine alone or in combination with statins would be more cost-effective, similar to findings based on Chinese pricing. This would provide an alternative for individuals with statin intolerance or serious side effects, or those preferring natural products, enabling them to pursue primary prevention.

We expect that the use of berberine, either alone or in combination with statins, will enhance the clinical guidelines and policies for primary CVD prevention. In 2018, the International Lipid Expert Panel (ILEP) suggested considering berberine for lipid-lowering intervention in patients who are intolerant to statins; however, the evidence at that time was limited^25^. The 2019 ESC/EAS guidelines on the management of dyslipidemia also acknowledged the lipid-lowering effects of dietary supplements and functional foods, including berberine^26^. Our findings demonstrate that berberine offers greater cost-effectiveness compared to statins when its price is moderate. This provides additional evidence for the use of berberine in patients intolerant to statins and supports the refinement of clinical guidelines and policies for CVD prevention.

This study has several notable strengths. First, the primary outcome was the occurrence of first CVD events or deaths, which provides a clinically relevant endpoint. The analysis also modeled the QALYs and costs throughout the lifetime, accounting for potential side effects of medications such as diabetes. This comprehensive approach offers a more complete picture of the cost-effectiveness of primary CVD prevention strategies. Additionally, the analysis considered the context-specific pricing of berberine. In the UK, berberine is priced as a high-cost nutritional supplement^19^. In contrast, berberine is widely used as a lower-cost drug in China due to its proven effectiveness^22^. Import tariffs and supply chain costs make UK prices unlikely to approach Chinese levels. However, scenario analysis using Chinese pricing data and deterministic sensitivity analysis (DSA) indicates that lower pricing could substantially improve the health economic viability of berberine-based interventions, underscoring the critical role of price. So, even partial international price reductions, without reaching Chinese price levels, would enhance the economic attractiveness of berberine as a primary prevention option.

However, the analysis also has several limitations. When estimating the cost-effectiveness of the combined intervention, the model parameters were derived from RCTs conducted in Chinese populations^37,38^. The sample sizes of these RCTs were relatively small, and the overall quality of the evidence was not optimal, which introduces potential bias into the final results. Currently, there is a paucity of high-quality RCT data evaluating the efficacy and safety of combined therapy approaches for managing hyperlipidemia and preventing CVD outcomes. The available evidence is primarily from the Chinese context. Likewise, we relied on RCTs with relatively small sample sizes for estimating berberine’s efficacy parameters. Additionally, the parameters in our model were primarily derived from the SHHEC, which was conducted mainly in the 1980s-1990s, where participants were predominantly aged 40-59 at recruitment^39^. In contrast, our target population for the cost-effectiveness analysis represents the contemporary Scottish adult population (aged 40 and older). This discrepancy in era and age structure may introduce a potential limitation. Another limitation of this study is the absence of high-quality randomized trials evaluating the effect of berberine on major cardiovascular events. Our analysis, therefore, relies on lipid-lowering as a surrogate, mapped to established cardiovascular risk equations. This exploratory use of surrogate-based modeling is consistent with early cost-effectiveness evaluations conducted for other lipid-lowering therapies, for example, initial CEA of PCSK9 inhibitors was performed before hard outcome trial results were available and was based solely on LDL-cholesterol reduction to estimate potential value^40^. So, our findings should be interpreted cautiously, as conditional and hypothesis-generating, pending future event-based trials. Furthermore, the Scottish dataset is not fully generalizable to the entire UK for cost‑effectiveness analysis due to the devolved nature of the healthcare system, which results in variation across nations in service organization, prescribing policies, and clinical pathways that influence resource use and costs^41^. Scotland also applies distinct hospital costing systems and unit cost frameworks compared with England and other UK nations, meaning Scottish costs cannot be directly transferred without re‑costing^42^. Historically, Scottish healthcare expenditures were higher than in the rest of the UK, indicating differences in resource utilization or underlying disease incidence^43^. Population characteristics such as socioeconomic deprivation, rurality, ethnicity, and baseline morbidity also differ between Scotland and the overall UK population, potentially affecting disease progression, healthcare utilization, and health‑related quality‑of‑life estimates^44,45^. While Scottish data can robustly inform clinical effectiveness, relative risks, and patterns of resource use, these differences limit the direct extrapolation of cost‑effectiveness results to the UK. Appropriate adjustments, re‑costing using UK relevant sources, and sensitivity analyses are therefore required to support generalizability. Despite these limitations, this study offers a valuable contribution by comprehensively modeling the lifetime cost-effectiveness of different intervention strategies, including combined interventions, to inform evidence-based decision-making for primary CVD prevention.

## Methods

### Study design

In this study, we estimated and compared the cost-effectiveness of different intervention strategies in a CVD-free population in Scotland from a Scottish healthcare payer perspective. The intervention strategies include no intervention, statins, berberine, and the combination of statins with berberine for primary prevention of CVD. Individual eligibility for statins, berberine or combined interventions is determined based on each individual’s 10-year CVD risk, which was estimated using the ASSIGN score^46^.

We selected Scotland as the study setting because the Scottish CVD Policy Model is an openly available, well-validated framework with population-specific data from the Scottish Health Survey (SHS), enabling robust and reproducible modeling. Although much of the existing evidence for berberine comes from trials in China, a European RCT has also demonstrated lipid-lowering efficacy in Caucasian participants^47^, supporting its relevance to UK populations, and we used this study when estimating efficacy. The lipid-lowering effect shown in this study (16.4% reduction in non-high-density lipoprotein cholesterol (non–HDL-c)) is similar to that in studies in Chinese (15.3% – 22.2% in non-HDL‑c)^20^. Moreover, berberine is widely available over the counter in the UK^19^, reinforcing the appropriateness of evaluating its potential economic impact in this context.

### Data sources and intervention-eligible population

We used data from the SHS to simulate the CVD-free population, which are publicly available from the UK Data Service and can be accessed at https://beta.ukdataservice.ac.uk/datacatalogue/ series/series?id=2000047. The SHS is a key national survey that collects comprehensive data on the health and well-being of the Scottish population. The SHS series was established in 1995, conducted annually with different focuses. Using a multi-stage stratified random sampling design, the SHS recruited adults aged 16 and over, as well as children aged 0–15 years, living in private households across 14 Health Boards in Scotland. The SHS collected data by questionnaire and nurse visits. The questionnaire covered information on individuals’ demographic characteristics, lifestyle behaviors, and the prevalence of various health conditions. The nurse visits covered the usage of medicines and supplements, physical examinations, blood sample, saliva sample and urine sample. Since the ASSIGN score implicated several risk factors, we finally included the SHS of 2003, 2008, 2009, 2010, and 2011, which have collected blood samples or recorded systolic blood pressure.

We obtained the eligible population for individuals aged 40 years or older without pre-existing CVD from the SHS dataset. This aligns with the recommendations from the National Institute for Health and Care Excellence (NICE) in England and Wales, which advises initiating CVD risk assessment in the population 40 years and above^48^. Those currently receiving statins were also excluded. Demographic characteristics and CVD risk factor data were obtained from the SHS. Participants with missing data on CVD risk factors (i.e., age, sex, diabetes, family history of CVD, daily cigarette usage, blood pressure, TC, HDL-c, and Scottish Index of Multiple Deprivation (SIMD)) were excluded as well. The final intervention-eligible population consisted of 3869 participants.

### ASSIGN score

The ASSIGN score is primarily used in the UK, and it is recommended as the preferred risk assessment tool by the Scottish Intercollegiate Guidelines Network (SIGN) for cardiovascular risk assessment in the UK. It is designed to provide a more accurate assessment of CVD risk compared to other risk assessment tools, such as the Framingham risk score, by taking into account socioeconomic status as an independent risk factor^49^.

For individuals without pre-existing CVD, preventive statin therapy is prioritized based on their estimated 10-year risk of experiencing a primary CVD event, such as a heart attack or stroke^48^. The ASSIGN score is a cardiovascular risk assessment tool that is used to estimate an individual’s 10-year risk of developing CVD, expressed as a percentage^46^. It was developed by the SIGN using data from the Scottish Heart Health Extended Cohort (SHHEC). The ASSIGN score takes into account several risk factors, including age, sex, family history of premature CVD, smoking status, blood pressure, diabetes, TC, HDL-c and SIMD.

Initially, the 10-year CVD risk threshold for statin eligibility was set at 20%. However, in 2014, the NICE in England and Wales recommended lowering the risk threshold for statin initiation to a uniform 10%^50^. In contrast, the SIGN continues to uphold a threshold of 20%^49^. Given the varying thresholds set by different guidelines^49,50^, our study used 10-year CVD risk thresholds of both 10% and 20% for intervention eligibility.

### The Scottish Cardiovascular Disease Policy Model

The Scottish CVD Policy Model is a comprehensive simulation model developed to inform primary prevention and management strategies in Scotland. This model aims to quantify the long-term health impact and healthcare cost of various interventions targeted at reducing the burden of CVD, which is suitable for our target.

The Scottish CVD Policy Model is an open-source, decision-analytic simulation model developed and validated using data from the Scottish population. Based on these ASSIGN risk profiles, which incorporate various individual-level risk factors, this model is able to project life expectancy, quality-adjusted life-years (QALYs), and healthcare costs for individuals receiving care within the Scottish NHS. The source code and programming files used to run the model are publicly accessible and can be downloaded at https://github.com/yiqiaoxin/CVDmodel.

We adapted the Scottish CVD Policy Model for our cost-effectiveness analysis. The core model structure of health states and transitions remained unchanged. Key adaptations included: (1) the addition of new treatment pathways for berberine and combined interventions; (2) selective updates to cost data of berberine, statin and combined interventions, while retaining original costs associated with clinical events; and (3) selective updates to efficacy variables of berberine and combination therapy, particularly concerning the parameters for the diabetes absolute risk increase, considering that statins may elevate diabetes risk^51^ while berberine reduce it^52^. All other model parameters and data sources remained consistent with the original model.

**Fig. 5:**
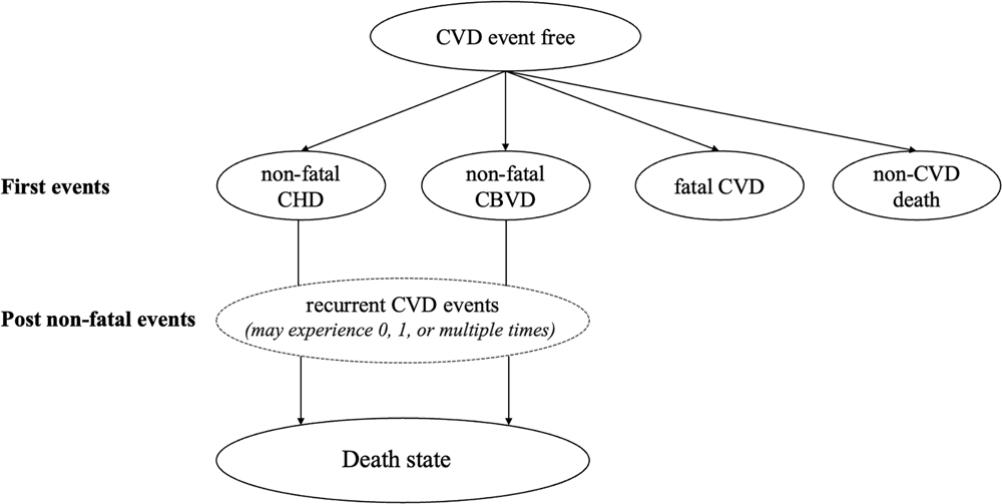
Structure of the Scottish Cerebrovascular Disease Policy Model. This diagram illustrates the schematic structure of health state transitions in the CVD Policy Model. CVD cardiovascular disease, CHD coronary heart disease, CBVD cerebrovascular disease.

Figure 5 shows a diagram of the state transition for this CVD Policy model. Individuals enter the model in a CVD‑free state and may transition to one of three CVD event states or to non‑CVD death. After experiencing a non‑fatal first CVD event, individuals move into the corresponding non‑fatal CVD state, where they remain at risk of experiencing recurrent CVD events in each subsequent model cycle until they ultimately transition to death.

CVD events were defined as both CVD cases and deaths diagnosed using ICD-9 codes 390-459 and ICD-10 codes G45, I00-I99. This includes rheumatic heart diseases, hypertensive diseases, ischemic heart diseases, pulmonary heart disease, other forms of heart disease, cerebrovascular diseases (CBVD), peripheral vascular diseases, and other circulatory system diseases. Specifically, non-fatal coronary heart disease (CHD) is defined by ICD-9 codes 410-414 and ICD-10 codes I20-I25, while non-fatal CBVD is defined by ICD-9 codes 430-438 and ICD-10 codes G45, I60-I69. Fatal CVD events are defined using ICD-9 codes 390-459 and ICD-10 codes I00-I99.

### Intervention strategies

We compared the cost-effectiveness of statins and berberine therapies by analyzing different intervention strategies in the SHS cohort, and included nine scenarios, including no intervention, atorvastatin 20 mg per day (statins), berberine 1000 mg per day (berberine), simvastatin 20 mg plus berberine 1500 mg per day (combined intervention 1) and simvastatin 20 mg plus berberine 900 mg per day (combined intervention 2) for individuals with an ASSIGN score ≥20% (ASSIGN ≥ 20%), and statins, berberine, combined intervention 1 and combined intervention 2 for individuals with an ASSIGN score ≥10% (ASSIGN ≥ 10%). Given the limited RCT data on the combination of statins and berberine, which have been evaluated solely in mainland Chinese cohorts^22^, we adopted the specific dose combinations from these trials: the study of He JJ et al.^38^. which used the dose combination of simvastatin 20 mg plus berberine 1500 mg, and the study of Su XY et al. ^37^. which used the dose combination of simvastatin 20 mg plus berberine 900 mg.

### Model variables

The key parameter values used in the Scottish CVD Policy Model, including state transition probabilities, intervention effectiveness, utilities, and cost inputs, are presented in Table 2 and Tables S4–S9.

**Table 2:** Intermediate-intensity intervention measures of statin, berberine and combined interventions

Parameters	Base case (95%CI)	Distribution	Data source	Reference
Intervention effectiveness				
Non-HDL-c reduction, %				
Atorvastatin 20 mg	26.0 (15.0–35.0)	Beta	Meta-analysis	^10^
Berberine 1000 mg	16.4 (14.6–18.2)	Beta	RCT	^47^
Simvastatin 20 mg plus berberine 1500 mg	34.4 (33.1–35.7)	Beta	RCT	^38^
Simvastatin 20 mg plus berberine 900 mg	44.6 (35.8–53.4)	Beta	RCT	^37^
HDL-c increase, %				
Atorvastatin 20 mg	4.0 (0.0–8.0)	Beta	Meta-analysis	^28,57^
Berberine 1000 mg	9.1 (3.8–14.4)	Beta	RCT	^47^
Simvastatin 20 mg plus berberine 1500 mg	25.0 (23.2–26.8)	Beta	RCT	^38^
Simvastatin 20 mg plus berberine 900 mg	17.4 (8.6–26.2)	Beta	RCT	^37^
Relative risk per 1.0 mmol/L reduction in non-HDL-c				
Nonfatal coronary heart disease	0.77 (0.75–0.80)	Beta	Meta-analysis	^10,28,58^
Nonfatal cerebrovascular disease	0.87 (0.83–0.91)	Beta	Meta-analysis	^10,28,58^
Fatal cardiovascular disease	0.90 (0.86–0.92)	Beta	Meta-analysis	^10,28,58^
Any cardiovascular disease	0.79 (0.77–0.81)	Beta	Meta-analysis	^10,28,58^
Side effects and intervention disutility				
Type 2 diabetes absolute risk increase, %				
Statins	0.50 (0.1–1.0)	Log-normal	Review	^28,51^
Berberine	−4.6 (−6.1 to −4.2)	Log-normal	Review&cohort	^51,52,59^
Combined statin and berberine	0.0 (−4.6–0.5)	Log-normal	Estimate	^51,52,59^
Annual pill-taking disutility	0.002 (0.000–0.004)	Beta	Estimate	^28,30^
Annual treatment-related costs, £				
Intervention cost				
Atorvastatin 20 mg/day	13.44 (8.99–23.99)	Gamma	Official tariff	^60^
Berberine 1000 mg/day	94.58 (27.34–424.86)	Gamma	Estimate	^19^
Simvastatin 20 mg plus berberine 1500 mg/day	150.86 (50.00–646.28)	Gamma	Estimate	^19,60^
Simvastatin 20 mg plus berberine 900 mg/day	94.11 (33.59–391.36)	Gamma	Estimate	^19,60^
Monitoring (appointments and blood tests), first year	102.51 (63.51–141.51)	Gamma	Official tariff	^28,50,60,61^
Monitoring (appointments and blood tests), subsequent	55.48 (16.48–94.48)	Gamma	Official tariff	^28,50,60,61^
Other costs, £				
Risk assessment	17.68 (10.68–24.68)	Gamma	Official tariff	^28,50,60,61^
Annual type 2 diabetes treatment	314.33 (156.50–469.50)	Gamma	Official tariff	^28,50,60,61^
Other costing parameters				
Annual discount rate, %	3.5 (1.5–5.5)	-	Estimate	^62^

*HDL-c* high-density lipoprotein cholesterol.

Risk assessment is conducted by calculating the ASSIGN score, which requires appointments and blood tests to collect the necessary data.

The state transition probabilities were derived through a competing risk parametric survival analysis based on two nationally recognized Scottish datasets: the SHHEC, a large population-based cohort with long-term follow‑up for cardiovascular risk factors and events, and the Scottish Morbidity Records (SMR)^53^, which provide comprehensive hospital admission and diagnostic data across Scotland. Together, these datasets capture incident CHD and CBVD events at the population level and allow estimation of age‑, sex‑, and risk‑factor-specific transition probabilities used in the model.

A parametric competing risks approach was used to estimate cause-specific hazard functions for the four primary events: non-fatal CHD, non-fatal CBVD, fatal CVD, and fatal non-CVD. Gompertz regression models were fit with the ASSIGN risk factors as covariates. Results for primary event regression, developed by Lewsey et al., are presented in Table S4^53^.

Transition probabilities from non-fatal events to all-cause mortality were similarly estimated using Gompertz regression. In this regression, age at first event, SIMD and family history of CVD were employed as covariates. Results for the secondary event regressions, also developed by Lewsey et al., are presented in Table S5^53^.

Additionally, the model accounts for the occurrence of secondary health events (e.g., angina, myocardial infarction) within the chronic disease states, with probabilities determined through probit regression on age at first event, SIMD, and family history of CVD (Table S6)^54^.

Each health state within the model is assigned a disutility value derived from a survey of the Scottish population^54^. Individuals without first CVD events are assigned age‑ and sex‑specific baseline utilities, while those entering non‑fatal CVD states incur event‑specific utility decrements. Additional utility losses may occur with recurrent CVD events.

QALY is a measure of the value of health outcomes by combining both the quantity (length of survival) and quality of life (health-related utility) into a single index^55^. It is calculated by multiplying life years gained by health utility weight. Health-related quality of life (HRQoL) inputs for the model were derived from the 2003 SHS, the most recent large-scale survey of the Scottish population to measure quality of life. A total of 7054 respondents aged 20 and older completed the 12-item Short Form (SF-12) HRQoL questionnaire, which was used to generate QALY values. Table S7 shows the mean HRQoL scores by age and socioeconomic group from the SHS 2003 dataset. These scores are used to weight survival probabilities across the model arms.

Linear regression was performed on the SHS 2003 data to estimate baseline QALY values for the Scottish adult population and to quantify utility decrements associated with various cardiovascular events. The dependent variable was the SF-12-derived QALY score, with independent variables including sex, age, and four CVD events. These regression results are presented in Table S8.

The healthcare costs associated with each health state were estimated using a combination of SMR data and national tariffs for elective and non-elective hospitalizations. Lifetime hospitalization costs were estimated for all individuals in the SHHEC-SMR dataset as a function of the events experienced and the corresponding length of stays. The costed SHHEC-SMR dataset was then analyzed to predict health state-related costs in the model.

The approach outlined by Geue et al. was used to attribute costs to each hospitalization episode in the dataset^56^. This involved assigning a healthcare resource group (HRG) to each episode using HRGv3.5 Grouper software, followed by applying the English NHS tariff. To avoid overestimating costs for continuous inpatient stays (CIS) involving multiple episodes, a ‘Spell Converter’ software was employed to designate a dominant episode for each CIS.

The estimated lifetime hospitalization costs for each individual were then used in linear regression models to predict pre- and post-event hospitalization costs for the model (Table S9). Cubic splines were included to capture non-linearity over time, and other covariates such as age at model entry (for pre-event costs), age at first event (for post-event costs), SIMD, and family history of CVD.

For ease of reference, we directly cited the numbers used in the study, which are available in the Tables S4–S9 and were originally published by Lewsey et al. ^54^.

### Intervention effectiveness

Statins and berberine reduced CVD risk by lowering individuals’ non–HDL-c levels. Statins have been shown to produce a 26.0% reduction in non-HDL-c^10,28,57^. Similarly, berberine has been found to decrease non-HDL-c by approximate 16.4% in the European population^47^. The combination use of statins and berberine has been shown to decrease non-HDL-c by approximate 34.4% (combined intervention 1)^38^ and 44.6% (combined intervention 2)^37^. Furthermore, meta-analytic evidence suggests that these reductions in non-HDL-c are associated with relative risks of 0.77 for non-fatal CHD, 0.87 for non-fatal CBVD, and 0.90 for fatal CVD per 1.0 mmol/L decrease in non-HDL-c^10,58^.

While evidence indicates that taking statins may increase the absolute risk of new-onset diabetes by 0.5%^51^, berberine has been shown to be effective in improving glucose metabolism and therefore decreasing diabetes risk by about 4.6%^52,59^. However, there is currently no evidence on the effects of the combined treatment of statins and berberine on diabetes, so we used the absolute risk of diabetes to be 0. Additionally, as previously^30^, an annual disutility of 0.002 QALYs was applied to account for the burden of daily pill-taking.

### Intervention costs

Statin costs were sourced from the British National Formulary^60^, and berberine costs were obtained from Amazon UK^19^. The cost of berberine was based on our latest search (9 Jan 2026) on Amazon (https://www.amazon.co.uk/s?k=berberine&ref=nb_sb_noss) using the keyword “berberine”. After sorting by “Featured” and excluding sponsored listings, we selected “WeightWorld Berberine (500 mg, 120 capsules)” as the base product, as it was the top-listed result and best-seller among single-ingredient options with a unit dose of ≤1500 mg. The price was £ 15.55 per bottle, yielding a unit cost of £ 0.026 per 100 mg. All individuals entering the model incurred screening costs. Regular checkups were assigned monitoring costs, which were largely derived from an analysis of unit costs for health and social care in England and Wales^48,61^. Furthermore, additional costs were incorporated for each statin or berberine user to account for the differential risk of developing diabetes associated with these interventions.

### Cost-effectiveness analysis

The Scottish CVD Policy Model was employed to assess the cost-effectiveness of different drug intervention strategies for individuals with ASSIGN ≥ 20%, as well as those with ASSIGN ≥ 10%. First, we assessed the cost-effectiveness of stains, berberine and combined interventions in comparison to no intervention. Next, we assessed the cost-effectiveness of berberine and combined interventions relative to statin therapy. The cost-effectiveness analysis commenced with a baseline simulation to predict health and cost outcomes in the absence of any intervention. Subsequently, the model was adapted to incorporate the benefits, side effects, and costs associated with statin or berberine therapy. Individuals from the SHS cohort were simulated, and if they met the intervention criteria, they were assigned the corresponding outcomes. Otherwise, no intervention effects were applied.

The study adhered to the Consolidated Health Economic Evaluation Reporting Standards (CHEERS) guidelines. We summed the predicted CVD events, QALYs, and costs across all individuals for each intervention strategy. The primary outcome was the incremental cost per QALY gained for the different intervention strategies, considering a lifetime horizon, using no intervention or statins as the reference. Intermediate outcomes, such as primary CVD events prevented, QALYs gained, and disaggregated costs, were also recorded with the same reference. Additionally, we calculated the number needed to treat (NNT) for each intervention, defined as the number of individuals who need to receive treatment to prevent one primary CVD event. Future costs and health benefits were discounted at an annual rate of 3.5%^62^. An intervention strategy was deemed cost-effective if its ICER was less than £20,000 per QALY, a standard threshold used in cost-effectiveness analyses in Scotland and the UK^63^.

We performed PSA and DSA to evaluate the uncertainty. The PSA was conducted using Monte Carlo Simulations. The parameter distributions and the risk factor hazard ratios in Table 2 were stochastically sampled. This allowed for the estimation of costs and QALYs for the respective intervention strategies in 1000 independent iterations. The base-case results were derived from the mean values of the probabilistic analyses. Additionally, 95% confidence intervals were calculated as the 2.5th and 97.5th percentiles of the 1000 iterations. Furthermore, cost-effectiveness acceptability curves were produced based on the results of the probabilistic analysis. The one-way DSA was conducted, and the lower and upper values used in these analyses were also detailed in Table 2. Results from the DSA were synthesized in tornado plots.

In Scotland, statins are available through the NHS as prescription medications, whereas berberine is classified as a nutritional supplement and is not covered, leading to significantly higher prices for berberine compared to statins^19,34,60^. This pricing structure may obscure the true cost-effectiveness of berberine. In contrast, in China, berberine is commonly used as a non-prescription medication, with a lower price that is comparable to that of statins^64,65^. Therefore, to assess the potential impact of differing regional drug costs, we conducted a scenario analysis using the prices of berberine and statins from China (Table S3). We obtained the statins and berberine costs from Chinese sources and repeated the entire analysis to determine if the cost-effectiveness results would change compared to the primary analysis using Scotland-based drug prices.

## Supplementary information

Supplementary Information

## Data Availability

Available from: ScotCen Social Research. (2019). Scottish Health Survey. [data series]. 2nd Release. UK Data Service. SN: 2000047, 10.5255/UKDA-Series-2000047.

## Code availability

The code used in this study is not publicly available but may be provided to qualified researchers upon reasonable request to the corresponding author.
