# Supplementary material for: Cost-effectiveness of statins, berberine, and combination for primary cardiovascular disease prevention in Scotland
Source: NPJ Cardiovasc Health. 2026 Apr 23;3:22. doi: 10.1038/s44325-026-00121-w (PMC13106774; doi:10.1038/s44325-026-00121-w)
Supplement: Supplementary file 1 — Supplementary Information [file 44325_2026_121_MOESM1_ESM.pdf]

Supplementary legends

Table S1 Comparison of CVD risk factors for the final intervention-eligible population.

Table S2 The cost-effectiveness of Statin, Berberine, and Combined Interventions in scenario analyses.

Table S3 Annual statin and berberine costs from Chinese source.

Table S4 Cause-specific hazards of primary first events

Table S5 Cause-specific hazards of post-CVD mortality

Table S6 Probit regression, probability of various secondary non-fatal CVD events within chronic disease states

Table S7 Mean (and 95% CI) HRQoL scores in the general Scottish population by age group and fifths of SIMD groups

Table S8 Event utility decrements

Table S9 Linear regression coefficient(95%CI), costs pre- and post-first events

Figure S1 Cost-effectiveness plane for all intervention strategies in scenario analyses.

The blue dashed line represents the cost-effectiveness threshold of £20,000/quality-adjusted life-year (QALY). An intervention strategy is considered cost-effective if the colorful point is below the blue dashed line.

Figure S2 Probabilistic sensitivity analysis for all intervention strategies in scenario analyses.

The blue dashed line represents the cost-effectiveness threshold of £20,000/quality-adjusted life-year (QALY). The proportions of dots below these blue dashed lines identify the likelihood that the Monte Carlo simulation results would yield an incremental cost-effectiveness ratio (ICER) below £20,000/QALY.

Figure S3 Cost-effectiveness acceptability curves for all intervention strategies in scenario analyses.

The blue dashed line represents the cost-effectiveness threshold of £20,000/quality-adjusted life-year (QALY). The probabilities of statins, berberine, and combined interventions being cost-effective are over 99% at a £20,000/QALY threshold.

Figure S4 Tornado plots for the influential model measures for all intervention strategies among individuals with ASSIGN risk ≥10%

The dashed line represents the baseline cost-effectiveness value of statins, berberine, and combined interventions.

Table S1. Comparison of CVD risk factors for the final intervention-eligible population

**Variables**	**Overall**	**Male**	**Female**	***p***
n	3869	1718	2151	
Age (years)	57.13 (11.88)	56.97 (11.82)	57.26 (11.94)	0.460
Survey year, n(%)				0.460
2003	2253 (58.2)	1006 (58.6)	1247 (58.0)	
2008	432 (11.2)	200 (11.6)	232 (10.8)	
2009	415 (10.7)	172 (10.0)	243 (11.3)	
2010	401 (10.4)	169 (9.8)	232 (10.8)	
2011	368 (9.5)	171 (10.0)	197 (9.2)	
SIMD groups (fifths), n(%)				0.651
1	764 (19.7)	337 (19.6)	427 (19.9)	
2	793 (20.5)	363 (21.1)	430 (20.0)	
3	861 (22.3)	394 (22.9)	467 (21.7)	
4	784 (20.3)	339 (19.7)	445 (20.7)	
5	667 (17.2)	285 (16.6)	382 (17.8)	
Diabetes, n(%)				0.278
No	3797 (98.1)	1681 (97.8)	2116 (98.4)	
Yes	72 (1.9)	37 (2.2)	35 (1.6)	
Family History of CVD, n(%)				0.298
No	3009 (77.8)	1350 (78.6)	1659 (77.1)	
Yes	860 (22.2)	368 (21.4)	492 (22.9)	
No. of cigarette per day(n)	3.37 (7.77)	3.63 (8.47)	3.17 (7.15)	0.064
SBP(mm Hg)	132.57 (18.66)	134.52 (16.56)	131.01 (20.05)	<0.001
TC(mmol/L)	5.92 (1.10)	5.79 (1.07)	6.03 (1.12)	<0.001
HDL-c(mmol/L)	1.54 (0.40)	1.38 (0.33)	1.66 (0.40)	<0.001

SIMD: Scottish Index of Multiple Deprivation; CVD: Cardiovascular Disease; SBP: Systolic Blood Pressure; TC: Total Cholesterol; HDL-c: High-Density Lipoprotein Cholesterol

Table S2. The cost-effectiveness of Statin, Berberine and Combined Interventions in scenario analyses

Intervention	Sample size	Number treated	Primary CVD events prevented	NNT	Undiscounted life- years	Discounted QALYs gained	Discounted cost, £	ICER, £/QALY
**Intervention strategies comparable to no intervention**								
Intervention eligibility: ASSIGN≥20%								
No intervention	3869	0	Reference	Reference	Reference	Reference	Reference	Reference
Statins	3869	1331	50.2 (30.5~68.0)	26.5	253.2 (94.4~428.3)	187.1 (60.6~328.9)	545276.5 (182708.1~894424.8)	2915.1 (2000.5~4191.8)
Berberine	3869	1331	42.1 (28.4~53.4)	31.6	206.6 (76.4~336.1)	149.3 (47.5~251.4)	177079.5 (-134437.8~457734.9)	1185.9 (-2534.8~1954.6)
Combined intervention 1	3869	1331	95.4 (63.9~120.9)	14	468.8 (160.2~764.3)	366.9 (124.9~598.1)	768624.8 (-10400.2~1498851.5)	2094.7 (-54.4~2636.7)
Combined intervention 2	3869	1331	100.6 (64.1~132.0)	13.2	499.8 (183.4~822.3)	391.3 (144.8~643.2)	625385.5 (-112349.8~1341214.5)	1598.1 (-684.3~2230.6)
Intervention eligibility: ASSIGN≥10%								
No intervention	3869	0	Reference	Reference	Reference	Reference	Reference	Reference
Statins	3869	2323	89.5 (55.1~121.7)	26	431.1 (163.5~722.1)	312.7 (99.0~546.9)	1506064.1 (789761.6~2198946.1)	4816.4 (3784.5~8221.0)
Berberine	3869	2323	75.7 (51.8~95.4)	30.7	355.3 (142.5~568.1)	250.6 (82.2~418.8)	691797.6 (117874.6~1219510.6)	2760.4 (1291.3~3249.5)
Combined intervention 1	3869	2323	171.8 (117.6~215.7)	13.5	803.9 (294.1~1296.1)	626.9 (222.5~1014.3)	2289701.8 (822266.9~3602499.5)	3652.5 (2971.9~4262.6)
Combined intervention 2	3869	2323	180.2 (118.0~234.3)	12.9	852.8 (331.9~1382.2)	665.7 (253.9~1083.8)	1980752.3 (569517.6~3369064.5)	2975.6 (1868.0~3380.8)
**Intervention strategies comparable to statin intervention**								
Intervention eligibility: ASSIGN≥20%								
Statins	3869	1331	Reference	26.5	Reference	Reference	Reference	Reference
Berberine	3869	1331	-8.1 (-15.6~-0.9)	31.6	-46.6 (-127.5~23.2)	-37.7 (-102.7~18.4)	-368197.1 (-510084.9~-239690.4)	9759 (-85437.8~68543.2)
Combined intervention 1	3869	1331	45.2 (31.7~55.0)	14	215.6 (13.0~393.4)	179.9 (19.8~320.2)	223348.2 (-253588.0~656548.8)	1241.6 (-6990.7~2270.8)
Combined intervention 2	3869	1331	50.4 (32.9~64.3)	13.2	246.6 (83.1~401.0)	204.3 (75.3~325.3)	80108.9 (-327556.4~468270.8)	392.1 (-3892.0~1482.0)
Intervention eligibility: ASSIGN≥10%								
Statins	3869	2323	Reference	26	Reference	Reference	Reference	Reference
Berberine	3869	2323	-13.8 (-27.1~-1.3)	30.7	-75.8 (-210.5~40.9)	-62.1 (-169.7~31.5)	-814266.5 (-1176726.3~-467051.7)	13117.5 (-54467.7~94062.6)
Combined intervention 1	3869	2323	82.3 (58.7~100.3)	13.5	372.8 (27.9~668.2)	314.2 (38.7~553.9)	783637.6 (-133960.4~1597051.4)	2494.2 (-1395.3~3089.4)

Combined intervention 2	3869	2323	90.7 (60.8~115.8)	12.9	421.7 (145.5~677.6)	353 (139.4~555.1)	474688.2 (-376654.5~1250230.8)	1344.8 (-3256.9~2259.6)

CVD: Cardiovascular Disease; NNT: the number needed to treat; QALY: quality-adjusted life-year; ICER: incremental cost-effectiveness ratio; ICER= Incremental cost / Incremental QALY

Table S3. Annual statin and berberine costs from Chinese source

**Parameters**	**Base case(95%CI)**	**Distribution**	**Data source**	**Reference**
**Intervention cost, £**				
Atorvastatin 20 mg/d	37.41 (8.11~43.99) (¥344.1(74.6~404.6))	Gamma	Estimate	64
Berberine 1000mg/d	26.99 (22.05~59.53) (¥248.2(202.8~547.5))	Gamma	Estimate	65
Simvastatin 20mg+ berberine 1500mg/d	47.15 (37.25~95.96) (¥433.6(342.6~882.6))	Gamma	Estimate	64,65
Simvastatin 20mg+ berberine 900mg/d	30.96 (24.04~60.25) (¥284.7(221.1~554.1))	Gamma	Estimate	64,65

Table S4. Cause-specific hazards of primary first events

Covariate	first_event_nonfatal_CHD	first_event_nonfatal_CBVD	first_event_CVD_death	first_event_nonCVD_death
**Male**				
age	1.046(1.039-1.053)	1.068(1.055-1.081)	1.097(1.085-1.108)	1.099(1.089-1.107)
SIMD	1.004(1.001-1.007)	1.009(1.005-1.014)	1.006(1.003-1.01)	1.009(1.007-1.012)
diabetes	1.921(1.339-2.757)	3.216(1.943-5.328)	2.37(1.476-3.808)	1.398(0.845-2.314)
Family History of CVD	1.504(1.343-1.685)	0.979(0.791-1.213)	1.179(0.997-1.394)	0.985(0.85-1.142)
No. of cigarette per day	1.018(1.013-1.022)	1.024(1.017-1.031)	1.031(1.026-1.038)	1.031(1.026-1.036)
Systolic Blood Pressure	1.008(1.005-1.011)	1.012(1.007-1.016)	1.015(1.012-1.019)	0.999(0.995-1.002)
TC	1.29(1.231-1.353)	1.087(0.998-1.183)	1.127(1.051-1.208)	0.95(0.895-1.008)
HDL	0.468(0.388-0.563)	0.882(0.674-1.155)	0.867(0.691-1.089)	1.468(1.239-1.74)
**Female**				
age	1.06(1.05-1.069)	1.083(1.067-1.1)	1.107(1.091-1.123)	1.095(1.084-1.106)
SIMD	1.009(1.006-1.012)	1.013(1.009-1.018)	1.004(1-1.009)	1.007(1.004-1.01)
diabetes	2.065(1.409-3.028)	3.007(1.813-4.988)	3.139(1.974-4.998)	0.964(0.513-1.811)
Family History of CVD	1.675(1.476-1.902)	1.428(1.162-1.754)	1.27(1.051-1.534)	0.982(0.848-1.138)
No. of cigarette per day	1.021(1.014-1.027)	1.027(1.017-1.038)	1.049(1.041-1.058)	1.039(1.033-1.045)
Systolic Blood Pressure	1.006(1.003-1.009)	1.014(1.009-1.018)	1.018(1.013-1.022)	1.003(0.999-1.006)
TC	1.207(1.146-1.271)	0.95(0.861-1.049)	1.059(0.978-1.146)	0.927(0.869-0.987)
HDL	0.474(0.392-0.574)	0.708(0.529-0.946)	0.84(0.657-1.076)	0.956(0.795-1.148)

CVD: Cardiovascular Disease; CHD: coronary heart disease; CBVD: cerebrovascular disease; SIMD: Scottish Index of Multiple Deprivation; TC: Total Cholesterol; HDL: High-Density Lipoprotein Cholesterol

Table S5. Cause-specific hazards of post-CVD mortality

Covariate	post_CHD	post_CBVD
**Male**		
Age at event	1.08(1.067-1.094)	1.069(1.049-1.091)
SIMD score	1.013(1.009-1.017)	1.009(1.003-1.015)
Family history	0.966(0.792-1.176)	1.063(0.769-1.47)
**Female**		
Age at event	1.077(1.061-1.093)	1.073(1.051-1.093)
SIMD score	1.007(1.003-1.012)	1(0.993-1.008)
Family history	0.752(0.595-0.95)	1.198(0.859-1.67)

CVD: Cardiovascular Disease; CHD: coronary heart disease; CBVD: cerebrovascular disease; SIMD: Scottish Index of Multiple Deprivation

Table S6. Probit regression, probability of various secondary non-fatal CVD events within chronic disease states

Covariate	Probit regression coefficient(95%CI)				
	CHD	Stroke	Intermittent Claudication	Other Heart Condition	Heart Failure
After first non-fatal CHD					
**Male**					
t1	-0.019 (-0.040-0.002)	-0.101 (-0.147--0.055)	-0.049 (-0.104-0.006)	-0.06 (-0.091--0.028)	-0.152 (-0.195--0.11)
t2	0.071 (0.045-0.098)	0.098 (0.035-0.161)	0.034 (-0.039-0.107)	0.05 (0.008-0.092)	0.16 (0.098-0.223)
Age at first event (years)	0.010 (0.005-0.016)	0.001 (-0.007-0.009)	-0.001 (-0.012-0.010)	0.005 (-0.002-0.013)	0.014 (0.004-0.023)
SIMD	0.003 (0.001-0.005)	0.003 (0.000-0.007)	0.002 (-0.003-0.006)	0.003 (0.001-0.006)	0.002 (-0.002-0.006)
Family history	0.106 (0.019-0.193)	-0.011 (-0.168-0.145)	-0.136 (-0.350-0.077)	0.174 (0.051-0.297)	0.044 (-0.121-0.21)

Constant	-2.012 (-2.420--1.605)	-2.085 (-2.640--1.530)	-2.137 (-2.903--1.371)	-2.187 (-2.68--1.693)	-2.626 (-3.26--1.992)
**Female**					
t1	-0.003 (-0.033-0.027)	-0.072 (-0.144-0.000)	-0.078 (-0.152--0.004)	-0.045 (-0.087--0.003)	-0.119 (-0.176--0.061)
t2	0.057 (0.011-0.10	0.026 (-0.102-0.155)	0.106 (-0.015-0.228)	0.026 (-0.045-0.096)	0.133 (0.039-0.227)
Age at first event (years)	0.010 (0.004-0.016)	0.007 (-0.006-0.019)	0.016 (-0.001-0.033)	0.012 (0.003-0.021)	0.018 (0.000-0.029)
SIMD	0.001 (-0.001-0.004)	0.005 (0.001-0.009)	0.001 (-0.005-0.008)	0.001 (-0.002-0.004)	0.004 (-0.000-0.009)
Family history	0.056 (-0.046-0.158)	-0.014 (-0.221-0.194)	-0.129 (-0.379-0.122)	-0.138 (-0.27--0.005)	-0.038 (-0.246-0.17)
Constant	-2.137 (-2.58--1.694)	-2.638 (-3.492--1.783)	-3.274 (-4.558--1.990)	-2.407 (-3.00--1.81)	-3.017 (-3.587--2.177)
After first non-fatal CBVD					
**Male**					
t1	-0.069 (-0.150-0.012)	-0.035 (-0.087-0.017)	0.020 (-0.085-0.125)	-0.070 (-0.134--0.006)	-0.129 (-0.275-0.017)
t2	0.063 (0.049--0.174)	0.046 (-0.030-0.123)	-0.108 (-0.305-0.09)	0.077 (-0.016-0.17)	0.159 (-0.021-0.340)
Age at first event (years)	-0.003 (-0.016-0.010)	0.010 (-0.001-0.021)	0.001 (-0.016-0.019)	0.004 (-0.009-0.017)	0.039 (0.009-0.070)
SIMD	-0.002 (-0.008-0.004)	0.003 (0.000-0.006)	0.007 (0.001-0.014)	0.002 (-0.002-0.007)	-0.010 (-0.021-0.001)
Family history	0.144 (-0.085-0.373)	0.019 (-0.154-0.191)	0.024 (-0.34-0.389)	0.053 (0.169-0.275)	0.353 (-0.063-0.77)
Constant	-1.506 (-2.42--1.605)	-2.109 (-2.891--1.327)	-2.744 (-4.034--1.454)	-1.923 (-2.789--1.058)	-4.697 (-6.983--2.41)
**Female**					
t1	0.078 (-0.028-0.183)	-0.023 (-0.077-0.03)	0.008 (-0.137-0.152)	-0.053 (-0.138-0.032)	-0.186 (-0.319--0.054)
t2	-0.088 (-0.225-0.049)	0.056 (-0.02-0.132)	-0.069 (-0.275-0.137)	0.034 (-0.071-0.140)	0.227 (0.069-0.386)
Age at first event (years)	-0.000 (-0.014-0.013)	0.022 (0.013-0.030)	-0.011 (-0.031-0.009)	0.006 (-0.005-0.017)	-0.002 (-0.017-0.014)
SIMD	0.004 (-0.003-0.011)	0.001 (-0.002-0.004)	0.000 (-0.013-0.014)	-0.001 (-0.006-0.004)	0.001 (-0.007-0.010)
Family history	0.068 (-0.222-0.359)	0.042 (-0.192-0.108)	-0.303 (-0.798-0.192)	0.281 (0.044-0.517)	0.036 (-0.36-0.432)

Constant	-2.531 (-3.653--1.409)	-2.777 (-3.424--2.130)	-1.714 (-3.543-0.115)	-2.178 (-2.983--1.373)	-1.881 (-3.219--0.542

CVD: Cardiovascular Disease; CHD: coronary heart disease; CBVD: cerebrovascular disease; t1: time spline 1; t2: time spline 2; SIMD: Scottish Index of Multiple Deprivation

Table S7. Mean (and 95% CI) HRQoL scores in the general Scottish population by age group and fifths of SIMD groups

Age group (years)	SIMD 1 (least deprived)	SIMD 2	SIMD 3	SIMD 4	SIMD 5 (most deprived)
Male					
35-44	0.834(0.816, 0.852)	0.838(0.820, 0.855)	0.823(0.805, 0.841)	0.811(0.787, 0.835)	0.777(0.744, 0.810)
45-54	0.825(0.805, 0.845)	0.827(0.806, 0.849)	0.808(0.783, 0.833)	0.791(0.756, 0.826)	0.762(0.729, 0.794)
55-64	0.845(0.826, 0.865)	0.803(0.780, 0.826)	0.820(0.796, 0.843)	0.782(0.751, 0.813)	0.718(0.680, 0.757)
65-74	0.813(0.784, 0.841)	0.822(0.791, 0.853)	0.802(0.775, 0.830)	0.761(0.729, 0.792)	0.732(0.697, 0.768)
75+	0.797(0.750, 0.843)	0.802(0.770, 0.835)	0.756(0.722, 0.791)	0.775(0.731, 0.818)	0.732(0.685, 0.779)
Female					
35-44	0.837(0.823, 0.852)	0.827(0.812, 0.841)	0.794(0.773, 0.815)	0.788(0.769, 0.808)	0.748(0.722, 0.774)
45-54	0.827(0.812, 0.843)	0.793(0.773, 0.812)	0.780(0.758, 0.802)	0.769(0.745, 0.792)	0.736(0.708, 0.764)
55-64	0.835(0.816, 0.854)	0.815(0.798, 0.832)	0.791(0.768, 0.814)	0.769(0.742, 0.796)	0.701(0.670, 0.732)
65-74	0.827(0.803, 0.851)	0.803(0.776, 0.830)	0.792(0.766, 0.818)	0.742(0.709, 0.776)	0.702(0.668, 0.736)
75+	0.741(0.702, 0.779)	0.765(0.732, 0.798)	0.715(0.681, 0.748)	0.693(0.655, 0.731)	0.689(0.652, 0.726)

SIMD: Scottish Index of Multiple Deprivation

Table S8. Event utility decrements

Covariate	Utility decrements (95%CI)

Male	
CHD	0.043 (0.019, 0.068)
Stroke	0.092 (0.061, 0.122)
Intermittent claudication	0.025 (-0.005, 0.056)
Other heart condition	0.043 (0.011, 0.074)
Female	
CHD	0.037 (0.007, 0.067)
Stroke	0.097 (0.067, 0.127)
Intermittent claudication	0.017 (-0.009, 0.043)
Other heart condition	0.023 (-0.011, 0.058)

CHD: coronary heart disease

Table S9. Linear regression coefficient(95%CI), costs pre- and post-first events

**Covariate**	**Pre-first-Event Costs, Linear Regression Coefficient(95%CI)**				**Post-first-Event Costs, Linear Regression Coefficient(95%CI)**	
	**Non-fatal CHD**	**Non-fatal CBVD**	**Fatal CVD**	**Fatal non-CVD**	**Post non-fatal CHD**	**Post non-fatal CBVD**
**Male**						
t1	18.6 (-2.0, 39.2)	5.5 (-38.0, 49.0)	26.1 (-7.1, 59.3)	42.1 (-4.5, 88.7)	-552.6 (-638.7, -466.6)	-680.0 (-854.7, -505.2)
t2	115.0 (70.3, 159.8)	156.6 (72.0, 241.2)	114.6 (56.8, 172.5)	237.2 (157.1, 317.4)	654.9 (554.5, 755.3)	787.7 (555.7, 1020)
Age at first event (years)	22.7 (16.4, 29.0)	17.3 (5.8, 28.9)	27.7 (16.5, 38.89)	24.7 (9.0, 40.5)	84.6 (66.9, 102.4)	112.6 (81.2, 144.0)
SIMD	5.2 (3.0, 7.4)	6.6 (2.9, 10.3)	3.8 (-0.1, 7.8)	4.7 (-0.5, 10.0)	14.2 (7.6, 20.8)	6.8 (-4.5, 18.1)

Family history	93.8 (1.5, 186.1)	-161.9 (-328.5, 4.8)	67.4 (-120.9, 255.7)	116.9 (-198.7, 432.6)	239.8 (-80.4, 560.0)	-102.2 (-717.2, 512.9)
Constant	-1121 (-1446, -795)	-832.8 (-1483, -182.4)	-1345 (-1981, -709.2)	-1029 (-1890, -169.2)	-1024 (-2107, 59.0)	-1836 (-4010, 338.3)
**Female**						
t1	-7.3 (-94.3, 79.8)	14.2 (-23.3, 51.6)	23.9 (-18.4, 66.2)	59.0 (11.5, 106.4)	-548.6 (-652.4, -444.8)	-542.3 (-744.1, -340.4)
t2	172.2 (-64.6, 408.9)	121.8 (57.7, 185.8)	144.7 (72.2, 217.3)	202.6 (125.8, 279.4)	745.4 (600.4, 890.3)	595.6 (357.4, 833.9)
Age at first event (years)	0.5 (-25.1, 26.2)	26.3 (15.0, 37.6)	33.7 (18.4, 49.0)	16.0 (-3.5, 35.4)	90.7 (68.5, 112.9)	97.1 (67.0, 127.2)
SIMD	10.6 (2.3, 19.0)	8.4 (2.4, 14.4)	5.5 (0.8, 10.2)	11.9 (5.6, 18.3)	13.6 (6.0, 21.3)	7.7 (-4.7, 20.0)
Family history	337.8 (-235.7, 911.4)	22.7 (-176.6, 222.0)	105.5 (-125.0, 336.0)	47.7 (-229.7, 325.2)	-227.9 (-596.5, 140.7)	-93.9 (-656.1, 468.4)
Constant	-214.2 (-1359, 930.8)	-1462 (-2123, -800.5)	-1727 (-2643, -809.9)	-832.0 (-1894, 230.1)	-1321 (-2900, 257.1)	-1251 (-3593, 1092)

CVD: Cardiovascular Disease; CHD: coronary heart disease; CBVD: cerebrovascular disease; t1: time spline 1; t2: time spline 2; SIMD: Scottish Index of Multiple Deprivation

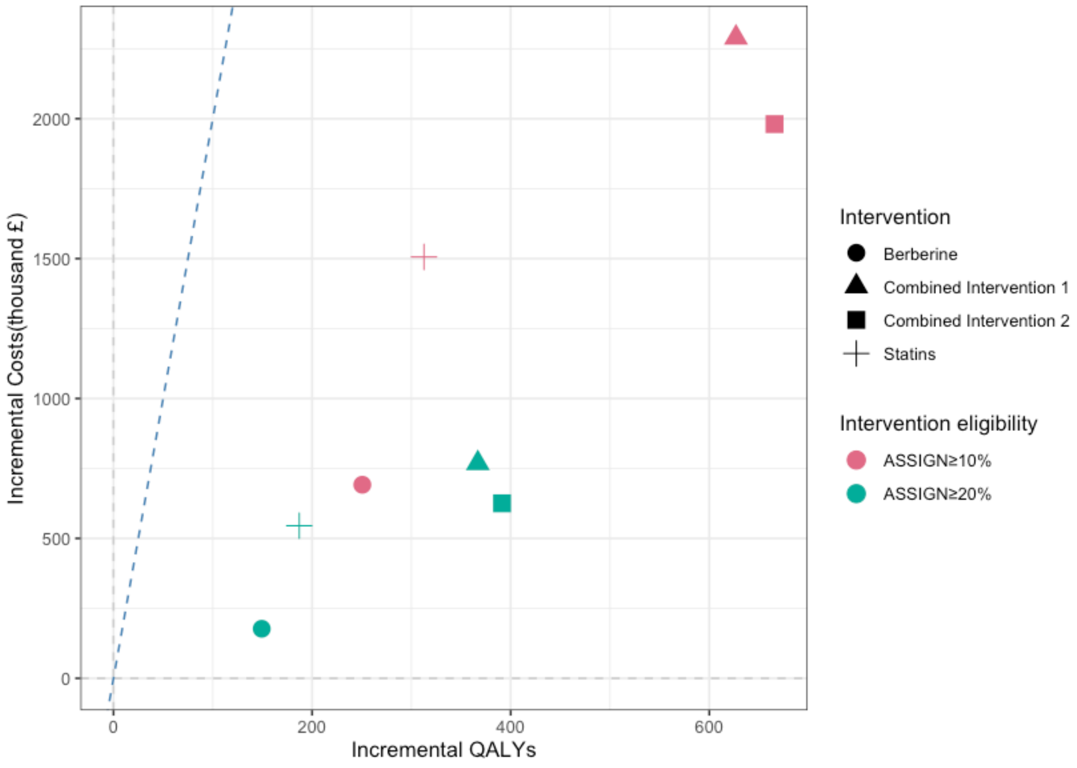

Figure S1 Cost-effectiveness plane for all intervention strategies in scenario analyses.

The blue dashed line represents the cost-effectiveness threshold of £20,000/quality-adjusted life-year (QALY). An intervention strategy is considered cost-effective if the colorful point is below the blue dashed line.

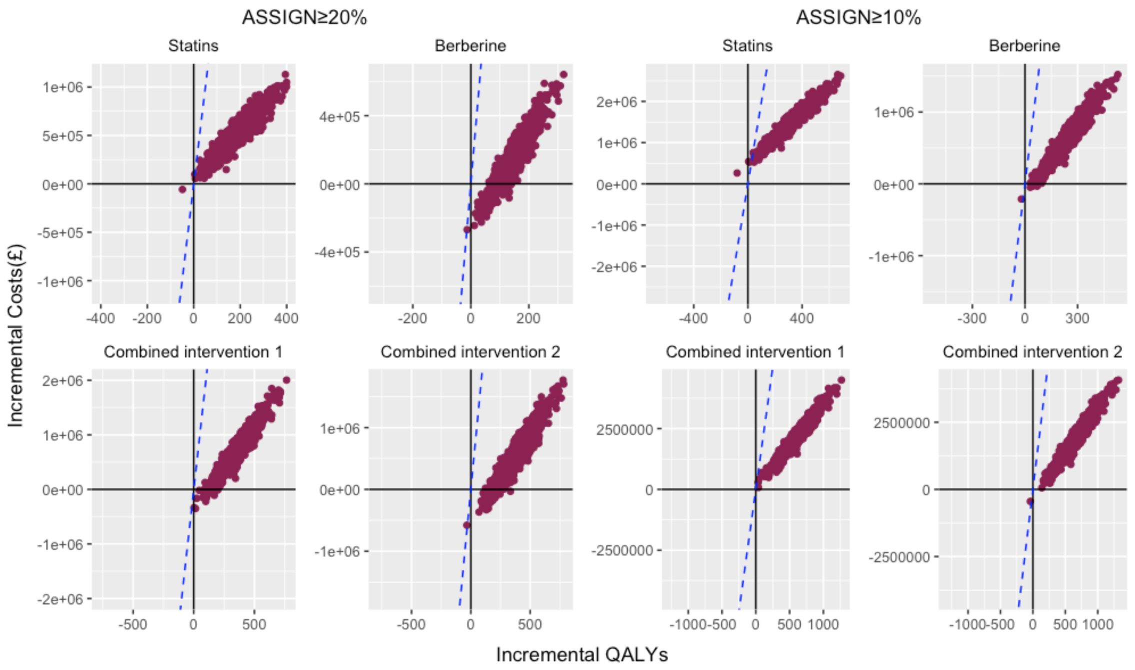

Figure S2 Probabilistic sensitivity analysis for all intervention strategies in scenario analyses.

The blue dashed line represents the cost-effectiveness threshold of £20,000/quality-adjusted life-year (QALY). The proportions of dots below these blue dashed lines identify the likelihood that the Monte Carlo simulation results would yield an incremental cost-effectiveness ratio (ICER) below £20,000/QALY.

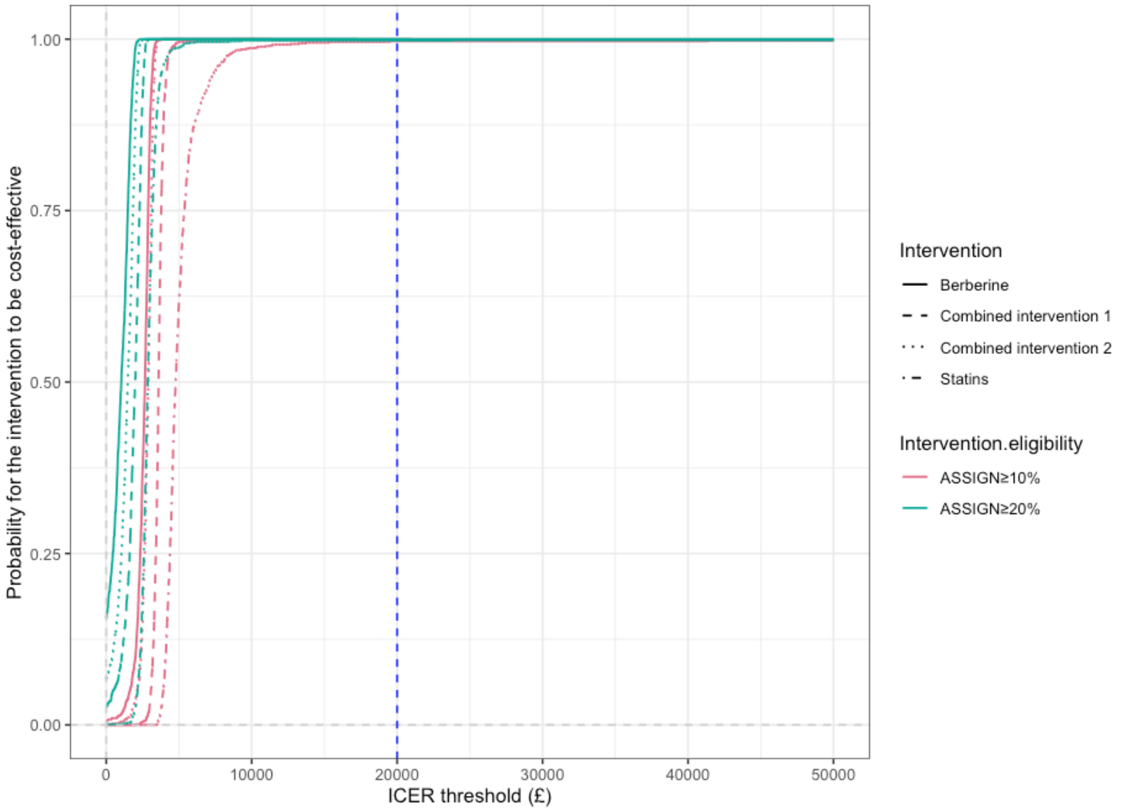

Figure S3 Cost-effectiveness acceptability curves for all intervention strategies in scenario analyses.

The blue dashed line represents the cost-effectiveness threshold of £20,000/quality-adjusted life-year (QALY). The probabilities of statins, berberine, and combined interventions being cost-effective are over 99% at a £20,000/QALY threshold.

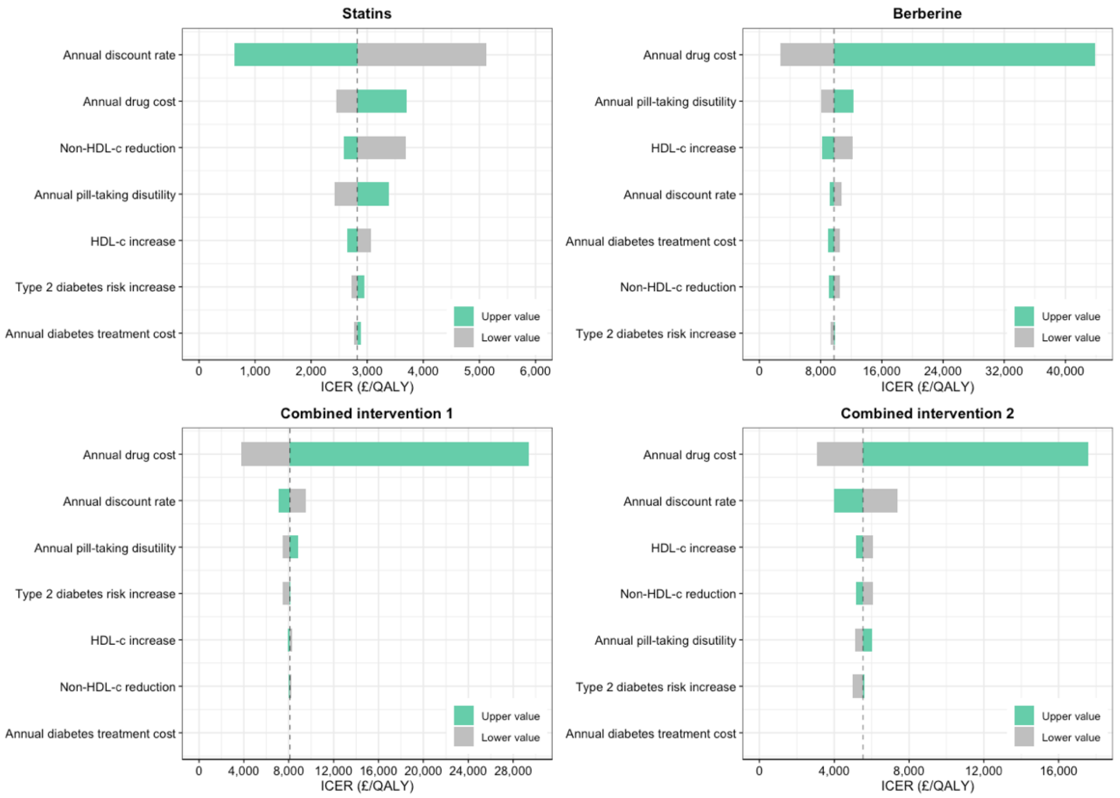

Figure S4 Tornado plots for the influential model measures for all intervention strategies among individuals with ASSIGN risk ≥10%

The dashed line represents the baseline cost-effectiveness value of statins, berberine, and combined interventions.
